# Validation of a Vitamin D Specific Questionnaire to Determine Vitamin D Status in Athletes

**DOI:** 10.3390/nu11112732

**Published:** 2019-11-11

**Authors:** D. Enette Larson-Meyer, Corey S. Douglas, Joi J. Thomas, Evan C. Johnson, Jacqueline N. Barcal, Jenna E. Heller, Bruce W. Hollis, Tanya M. Halliday

**Affiliations:** 1 Department of Family and Consumer Sciences, University of Wyoming, Laramie, WY 82071, USA; coreydouglas25@gmail.com; 2 Division of Kinesiology & Health, University of Wyoming, Laramie, WY 82017, USA; ejohns54@uwyo.edu; 3 Lake Erie College of Osteopathic Medicine, Erie, PA 16509, USA; 4 University of Minnesota Athletics, University of Minnesota, Minneapolis, MN 55455, USA; thomasjj@umn.edu; 5 IMG Academy, Bradenton, FL 34210, USA; Jackie.Barcal@img.com; 6 Wellness Department, Arizona State University, Tempe, AZ 85287, USA; jenna.e.heller@gmail.com; 7 Dr Bruce Hollis’ Laboratory, Medical University of South Carolina, Charleston, SC 29425, USA; hollisb@musc.edu; 8 Department of Health, Kinesiology, and Recreation, University of Utah, Salt Lake City, UT 84112, USA; tanya.halliday@utah.edu

**Keywords:** vitamin D intake, vitamin D deficiency, semi-quantitative food frequency questionnaire, FFQ, sun exposure

## Abstract

The study objective was to validate a food frequency and lifestyle questionnaire (FFLQ) to assess vitamin D intake and lifestyle factors affecting status. Methods: Data collected previously during the fall (*n* = 86), winter (*n* = 49), and spring (*n* = 67) in collegiate-athletes (Study 1) and in active adults (*n* = 123) (Study 2) were utilized. Study 1: Vitamin D intake and ultraviolet B exposure were estimated using the FFLQ and compared to serum 25(OH)D concentrations via simple correlation and linear regression modeling. Study 2: Vitamin D intake from food was estimated using FFLQ and compared to vitamin D intake reported in 7-Day food diaries via paired t-test and Bland–Altman analysis. Results: Study 1: Serum 25(OH)D was not associated with vitamin D intake from food, food plus supplements, or sun exposure, but was associated with tanning bed use (r = 0.39) in spring, supplement use in fall (r = 0.28), and BMI (body mass index) (r = −0.32 to −0.47) across all seasons. Serum 25(OH)D concentrations were explained by BMI, tanning bed use, and sun exposure in fall, (R = 0.42), BMI in winter (R = 0.32), and BMI and tanning bed use in spring (R = 0.52). Study 2: Estimated Vitamin D intake from food was 186.4 ± 125.7 via FFLQ and 148.5 ± 228.2 IU/day via food diary. There was no association between intake estimated by the two methodologies (r = 0.12, *p* < 0.05). Conclusions: FFLQ-estimated vitamin D intake was not associated with serum 25(OH)D concentration or food-record-estimated vitamin D intake. Results highlight the difficulty of designing/utilizing intake methodologies for vitamin D, as its status is influenced by body size and both endogenous and exogenous (dietary) sources.

## 1. Introduction

Vitamin D plays an important role in the health of active individuals and athletes and may influence performance via its impact on immune function, inflammatory modulation, bone health, and skeletal muscle function [1]. Vitamin D can be synthesized endogenously in the skin as a result of exposure to ultraviolet B radiation (UVB, 280–315 nm) from sunlight, or obtained in the diet from limited foods including fatty fish, egg yolks, and in many countries, fortified milk, margarine, citrus juice, and ready-to-eat breakfast cereals [2]. Endogenous synthesis is thought to account for the majority of vitamin D(25(OH)D) in circulation during the warmer, sunny months (spring, summer, and fall) [3]. A period of 5 to 30 minutes (depending on time of day, season, latitude, and skin pigmentation) of sunlight exposure to the arms and legs, twice weekly between the hours of 10 AM and 3 PM is generally considered sufficient to maintain adequate status. Supplemental vitamin D also serves as an important, readily-available source [3,4]. Despite this, vitamin D deficiency is common among the general population worldwide [5] and among some athletic populations [1,6]. Studies in athletes have shown that the prevalence of vitamin D deficiency (serum 25(OH)D concentration < 20 ng/mL) is highly variable and ranges from 2.4% [7] to 91% [8] in athlete groups tested. This risk of deficiency is influenced by season [7,8,9], training location [7,8,10], sport [7], and the athletes’ skin pigmentation [8] and body composition [11], all of which influence endogenous vitamin D synthesis [2]. Season, timing of exposure, altitude, air pollution, and geographical latitude influence how much UVB radiation reaches the Earth’s surface [12,13], whereas sunscreen use and clothing block UVB exposure to the skin [14,15]. Sufficient UVB radiation for vitamin D synthesis only occurs close to solar noon (i.e., between ~10 AM and 3 PM) during late spring, summer, and early fall months at latitudes greater than 33 to 35 degrees north or south [12,16]. Dietary sources of vitamin D are thought to have only a small effect on serum 25(OH)D concentration [2,17], but become more important when UVB exposure is insufficient [2]. Thus, the combination of limited dietary sources and the limitations of endogenous synthesis influence vitamin D deficiency as well as insufficiency (serum 25(OH)D concentration < 30 but > 20 ng/mL) 

Given the high prevalence of vitamin D insufficiency and deficiency and its potential to influence athlete health and sports performance, it is important to have tools to assess vitamin D intake and the lifestyle factors that affect vitamin D status. Obtaining accurate estimates of intake is particularly important in athletes with minimal sun exposure due to sport, training location or personal preference, as it helps determine both the etiology of insufficient vitamin D status and the appropriate intervention [1]. Food frequency questionnaires (FFQ) are often used in the clinical, sports nutrition/medicine and research settings to assess vitamin D intake and help assess vitamin D status. FFQs are particularly useful when assessing status of nutrients with a limited number of rich dietary sources, such as vitamin D [18], and can be easily and cost-effectively administered to athletes with minimal respondent demand [18]. A general FFQ alone, however, may not be sufficient to evaluate vitamin D status or predict those at risk for deficiency/insufficiency. A vitamin D-specific FFQ that also includes lifestyle factors (that have the potential to influence vitamin D status) including time spent in the sun, tanning bed use, and sunscreen use was developed for use in athletic populations [7]. Despite its use in previous studies from our group [7,11,19] and others [20,21,22], validation of our questionnaire has not been conducted. Several previous studies have evaluated vitamin D-specific FFQs in elderly [23,24], young [25,26], and ethnically diverse [27] populations with mixed results, however, validation studies in athletes and active populations are lacking, as are studies evaluating FFQs that also include lifestyle components. The purpose of this study was to attempt to validate/cross-validate vitamin D-specific food frequency and lifestyle questionnaire (FFLQ) [7] for use in athletic groups using biomarkers of status and 7-day food records (or diaries). A secondary purpose was to determine which FFLQ-estimated UV exposure variables were predictive of vitamin D status in the fall, winter, and spring. 

## 2. Materials and Methods 

This study was conducted using data collected previously in collegiate-athletes using 25(OH)D as a biomarker of vitamin D status (Study 1), and in healthy active individuals using vitamin D intake estimates collected via 7-day food diaries as the intake marker (*Study 2*). Participants on both studies resided at 41.3° N latitude and at 2195 m above sea level. The original studies were approved by the institutional review board at UW; participants had provided written, informed consent as to the procedures, associated risks, and future use of their data for related analyses. 

### 2.1. Study 1: Biomarker Validation 

#### 2.1.1. Study Population

Data from student-athletes was collected from September 2008 to March 2015 as part of four separate studies evaluating the vitamin D status of college athletes in general [7], and collegiate wresters [19], swimmers, and basketball players (unpublished data). Data for all athletes who; a) had completed the FFLQ; b) had simultaneous measurements of serum 25(OH)D; and c) had basic anthropometric data collected during the fall (late September/early October), winter (late January/early March), and/or spring (late April/early May) seasons, were included in the analyses. 

#### 2.1.2. Food Frequency and Lifestyle Questionnaire

The vitamin D–specific FFLQ (Appendix A) focused on dietary and lifestyle habits that potentially influence vitamin D status. The questionnaire [7] evaluates the frequency of consumption of vitamin D-containing foods and supplements. Frequency was evaluated according to the following: a) Never or less than one time per month; b) one to three times per month; c) one time per week; d) two to four times per week; e) five to six times per week; f) one time per day; g) two to three times per day; h) four to five times per day; i) six or more times per day). The vitamin D-containing foods originally included 15 foods/beverages (cows’ milk, soy or rice milk, eggs, vitamin D–fortified cereal, margarine, Subway sandwich bread, orange juice, and fatty fishes) and other vitamin D-fortified food category; and after the fall of 2014 included several additional vitamin D-fortified foods (e.g. Yoplait yogurt, Rockin’ Refuel) available on the athletes’ refueling station. Intake of vitamin D was estimated by multiplying the frequency midpoint by the average content of each vitamin D–containing food and expressed in International Units (IU) per day (assuming 30 days per month). The vitamin D content of foods was obtained from the USDA national nutrient database for standard reference [28], the data of Chen et al. [29], and selected food labels. Vitamin D intake from supplements was gathered in a separate section of the FFLQ. This section contained five questions and addressed multivitamin use and use of vitamin D, calcium, and vitamin D plus calcium supplements, and offered the option of including specific brand(s) and supplement doses if known. The amount of vitamin D from supplements was calculated using 400 IU (before 2011) or 600 IU as a standard for a multivitamin and 1000 IU as a standard for supplemental vitamin D, unless the actual dose or specific brand was reported. The FFLQ also addressed frequency of leisure time spent outside in the sun between 10 AM and 3 PM, frequency of tanning bed use, type and frequency of sunscreen applied (never, sometimes, usually, or always), and type of clothing typically worn outdoors. Total sun exposure was estimated from the sum of reported leisure time spent outdoors and tanning bed use. 

#### 2.1.3. Serum 25(OH)D Concentration/Vitamin D Status

A 5 mL blood sample was collected in standard serum tubes, allowed to clot for 30–60 minutes at room temperature, and centrifuged at 3500 rpm for 15 minutes. Aliquots of serum were stored at –20°C and later analyzed for 25(OH)D concentration using Diasorin 25(OH)D radioimmunoassay (Bruce Hollis’ Laboratory, the Medical University of South Carolina, Charleston, SC, USA). Vitamin D deficiency was defined as a serum 25(OH)D concentration < 20 ng/ml, whereas insufficiency was defined as a serum 25(OH)D concentration < 30 ng/mL but > 20 ng/mL [3]. Optimal status was considered a serum 25(OH)D ≥ 40 ng/mL [3], which is the lower limit achieved by humans living naturally in sun rich environments [30].

#### 2.1.4. Statistical Analysis

Statistical analyses were conducted using IBM SPSS Statistics Version 24 (SPSS Inc., Chicago, IL). Spearman Rank Correlation Coefficients were used to determine correlations between serum 25(OH)D concentration and variables collected by the FFLQ and anthropometric assessment, including vitamin D intake from food, vitamin D intake from supplements, total vitamin D intake from food and supplements, leisure time spent outside, tanning bed use, total UV exposure, sunscreen use, and body mass index (BMI). Spearman Rank Coefficients were used instead of Pearson Correlation Coefficients due to the categorical-nature of the FFLQ data and the general non-normal distribution of the data. Backward linear regression was used to create models to explain predictors of serum 25(OH)D using potential prediction variables (vitamin D intake from food and supplements, tanning bed use, time spend outdoors), and BMI. One-way ANOVA was used to test for differences in vitamin D intake, status, and sunlight exposure by season. 

### 2.2. Study 2: Food Diary Cross-Validation

#### 2.2.1. Study Population

The study was conducted using baseline data collected on 123 male and female volunteers that were part of a study evaluating urine color as a marker of change in daily water intake [31]. Participants aged 18–45 years were recruited through ListServ e-mail announcements, and flyer placement around Laramie, Wyoming. The study recruited only subjects who were generally active and were determined to be healthy. Individuals who made any dietary changes in the past month or who engaged in excessive exercise (> 4 h/week) were excluded from the study. Data for all subjects who; (a) completed the vitamin D questionnaire; b) completed a 7-day food diary, and (c) had basic anthropometric data were included in the analyses.

#### 2.2.2. Food Frequency and Lifestyle Questionnaire

The FFLQ (Appendix A) was completed at baseline. The FFLQ was an updated version of the FFLQ used in study 1 that incorporated additional vitamin D-rich fish/seafood sources (haddock, halibut, herring, perch, seabass, swordfish, light tuna, clams, lobster, oysters, scallops, and shrimp). Vitamin D intake from food alone and food plus supplements was calculated, but only intake from food was compared to intake reported on the food diaries.

#### 2.2.3. Food Diaries

7-day food diaries were obtained from participants in the week immediately after completion of the FFLQ. Participants were instructed to record all the foods and fluids consumed over the 7-day period, including the food preparation method, brand name (as applicable), and portion consumed in household or package units (See Supplemental File). Participants were asked to avoid specific dietary supplements during this period, but otherwise not to deviate from their typical dietary habits. The list of excluded supplements was: Calcium, chromium, vitamin C, cat’s claw, chaparral, cranberry, creatine, ephedra, germanium, hydrazine, licorice, l-lysine, pennyroyal, thunder god vine, willow bark, wormwood oil, yellow oleander, and yohimbe. Vitamin D intake was estimated using the 2015 Nutrition Coordinating Center (NCC) Nutrient Data System for Research (NDSR, version 2015, University of Minnesota, Minneapolis, MN). This database uses the USDA Nutrient Data Laboratory as the primary source of food nutrient composition, but is also supplemented by food manufacturers’ information and data available in the scientific literature. Questions that subjects had about the record-keeping procedures were clarified by the investigative team, and all nutrient data collected from the food diaries were compiled by a single team member (upper-level dietetic student) overseen by a Registered Dietitian (D.E.L.M.). 

#### 2.2.4. Statistical Analysis

Statistical analyses were conducted using IBM SPSS Statistics Version 24 (Chicago, IL.) A paired t-test was used to compare vitamin D intake estimated from the FFLQ, and intake estimated using 7-day food diaries and the coefficient of variation (CV) calculated between each day of the 7-day diaries. The Bland–Altman method combined with linear regression was used to assess the agreement between vitamin D intake estimated from the FFLQ and the diaries. In these analyses, differences in estimated vitamin D intake between the FFLQ and food diaries were plotted and compared against average estimated vitamin D intake from both dietary intake methodologies to evaluate differences in methods across the range of intakes, with a 95% CI calculated as limits of agreement. 60 IU (10% U.S. Recommended Dietary Allowance, RDA) was considered to be good estimation between measurements.

## 3. Results

### 3.1. Study 1: Biomarker Validation

#### 3.1.1. Physical Characteristics and Vitamin D Status

The characteristics of the athletes who participated in the study during fall, winter, and spring seasons are summarized in Table 1. Serum 25(OH)D concentrations varied across the seasons (*p* < 0.0001) and were highest in fall. In the fall, 15.1% of athletes (*n* = 13) were vitamin D insufficient and 4.7% (*n* = 4) had a 25(OH)D concentration indicative of vitamin D deficiency (25(OH)D ≤ 20 ng/mL). In the winter, 53.1% (*n* = 26) of athletes were vitamin D insufficient and 14.3% (*n* = 7) were vitamin D deficient, whereas 26.9% (*n* = 18) were insufficient and 13.4% (*n* = 9) were deficient in the spring. Using the cutoff of 40 ng/mL [30], 54.7% (*n* = 47), 10.2% (*n* = 5), and 23.9% (*n* = 16) of athletes had optimal vitamin D status in the fall, winter, and spring, respectively. Vitamin D status was correlated with BMI in all three seasons (*p* < 0.05, Table 2).

#### 3.1.2. Vitamin D Intake and Association between Intake and Status

FFLQ-estimated vitamin D intake from food, supplements, and food plus supplements are summarized in Table 1. FFLQ-estimated vitamin D intake from food (*p* = 0.019) and total vitamin D intake (food plus supplements combined) (*p* = 0.012) varied across seasons, but intake from supplements alone did not (*p* = 0.126). Few athletes obtained the US RDA for vitamin D (600 IU/day) from food alone (14% in fall, 32.7% in spring, and 14.9% in winter) but this percentage was higher when supplementary vitamin D along with food was taken (46.5%, 32%, and 28.4% in fall, winter, and spring, respectively). Vitamin D status was not directly associated with vitamin D intake from food, or food plus supplements during any season (Table 2). Vitamin D status, however, was associated with intake from supplements during the Fall (r = 0.280, *p* = 0.009) but not during the winter or spring. 

#### 3.1.3. UV Exposure and Association between Vitamin D Status and UV Exposure 

Leisure time spent outside in the sun, tanning bed use, and total UV exposure are summarized in Table 1. There was a trend for reported leisure time sun exposure (*p* = 0.062) and total UV exposure (*p* = 0.072) to be higher in the fall; seasonal differences were not observed for tanning bed use (*p* = 0.25). 

Serum 25(OH)D concentrations were not associated with reported leisure time spent outside in the sun or calculated total UV exposure during any season, but reported tanning bed use was associated with vitamin D status in the spring (Table 2). Sunscreen use was positively associated with vitamin D status in the fall and spring, but not during the winter.

#### 3.1.4. Predictors of Serum 25(OH)D

Backward linear regression models of serum 25(OH)D predictors are shown in Table 3. In the fall, BMI, tanning bed use, and outside sun exposure predicted vitamin D status (Model R = 0.424). In the winter, only BMI was a predictor (R = 0.315), and in the spring, BMI and tanning bed use were predictors (R = 0.516.) Total or supplemental vitamin D intake was not a predictor of status despite forced enforced entry into backward regression models. 

### 3.2. Study 2: Food Diary Cross-Validation

#### 3.2.1. Physical Characteristics and Vitamin D Intake

Characteristics of the 123 active participants and vitamin D intake estimated from the FLFQ and 7-day food diaries are shown in Table 4. 21 participants (17%) reported taking supplemental vitamin D as multivitamin or vitamin D supplement in the past three months. Vitamin D intake was highly variable across the seven days of collection on the food diaries; the coefficient of variation across all days averaged 79.5 ± 33.7% (range = 1.7 to 172%). None of the participants met the RDA for vitamin D intake according to FFLQ estimates, and only 2 (1.6%) met it according to the 7-day food diary. 

#### 3.2.2. Association and Difference between Vitamin D Intake Estimates

**Figure 1:**
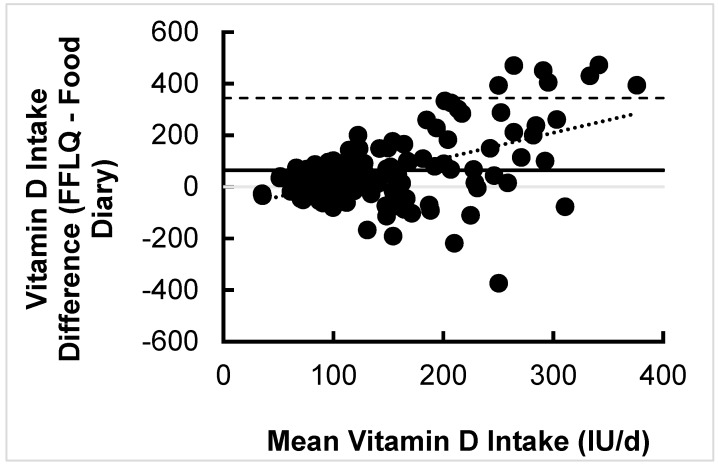
Bland–Altman plot demonstrating the association between average vitamin D intake from food sources estimated from the FFLQ and 7-day food diaries and the difference between both methods. The mean difference = 64.2 IU, with dashed lines illustrating the upper and lower limit of agreement after adding a ±1.96-fold standard deviation.

There was a trend for intake estimated using the FFLQ to be higher than that estimated from the 7-day food diaries (*p* = 0.099) with an absolute difference of 37.9 ± 253.0 IU. Two individuals had average vitamin D estimates in excess of 1500 IU/day from the food diaries (compared with 346 and 169 IU via the FFLQ, respectively); these data were obvious outliers on a simple scatterplot and the Bland–Altman plot and were therefore removed from further analysis. There was no association between vitamin D intake estimated from the FFLQ and food diaries whether all participants were included (r = 0.12, *p* = 0.21, *n* = 123) or the outliers removed (r = 0.10, *p* = 0.29, *n* = 121). The Bland–Altman analysis with outliers removed (Figure 1, *n* = 121) identified a positive association between the mean and the difference of vitamin D estimated using the FFLQ and diaries (*p* < 0.0001) with an R^2^ value of 0.2592. A significant relationship between these variables indicates that the Bland–Altman analysis is skewed (i.e., as the mean IU increases as the differences between the mean increases. The interval from –215.8 IU (lower agreement limit) to 344.2 IU (upper agreement limit) was obtained for the limit of agreement after adding a ±1.96-fold standard deviation. 

## 4. Discussion

The original intent of this two-part study was to validate a vitamin D food frequency and lifestyle questionnaire (FFLQ) that takes into account vitamin D-rich foods, supplements, and lifestyle factors that impact vitamin D status in athletes and active individuals. Study 1 used serum concentration of 25(OH)D as a means of validating the FFLQ-estimated vitamin D intake and UVB exposure, and Study 2 used 7-day food diary-estimated vitamin D intake to cross-validate the FFLQ estimates. The study was unable to find direct correlation between the FFLQ-estimated vitamin D intake and either vitamin D intake from a 7-day food diary or serum 25(OH)D concentration as the biomarker of vitamin D status. Several FFLQ-estimated UV exposure variables were predictive of vitamin D status in the fall and spring in regression models that control for BMI. These results highlight the difficulty of questionnaire validation for a vitamin whose status is influenced by body tissue stores and endogenous and exogenous sources. 

Previous studies have utilized biomarkers of serum 25(OH)D concentration, diet history methodologies, or both in order to validate vitamin D-specific FFQs in various populations of non-athletes. A consistent finding among published studies is that vitamin D intake estimates by FFQs tend to average 3.2% [32] to 84% [24] higher than intake estimates calculated by reference methods, including food diaries obtained over 3 to 14 days [23,24,25,32,33]. In the present study, intake estimated by the FFLQ also tended to be higher (averaging 38 IU/day or 25.6% higher) than intake estimated using 7-day food diaries. Considerable variation, however, was observed among individuals with both over- and underreporting by the FFLQ relative to the food diaries. 

Differences between estimates of vitamin D intake using our FFLQ compared to food diaries and a lack of correlation between both methods, however, does not necessary imply that our FFLQ is invalid. Rather, in the absence of a gold standard for dietary assessment [34], such differences likely reflect errors in both methodologies, as well as differences in the recalled/recorded collection period (i.e., 3 months vs. 7 days, neither of which is reflective of the half-life of serum 25(OH)D [35]). As vitamin D is found in a limited array of foods consumed with variable frequency [32] (e.g., fatty fishes, fortified products), capturing estimates of typical intake can be challenging via all methodologies because a certain length of time is required to account for daily intra-individual variation. As such, intake by food diary would be lower than that estimated by the FFQ if infrequently consumed foods were not eaten during the 7-day window when the food diary was completed, or vice versa if such foods were consumed with greater frequency. Additionally, under-reporting is a known disadvantage of food diary methodology [18] that may pertain to vitamin D as well as energy intake. Results from the Bland–Altman analysis (Figure 1) in the present study highlight both the degree of variability among the FFLQ and food diaries and a significantly positive mean bias (upward trend), which is suggestive of more error with higher mean intakes. The lines of agreement of the Bland–Altman plot, which were 560 IU apart (nearly the RDA), further highlight that differences between the FFLQ and food diary estimates were inconsistent amongst participants. 

The semi-quantitative and self-administered design of our FFLQ questionnaire may also have influenced the accuracy of our vitamin D intake results. The FFLQ provided standard portion sizes rather than asking participants to record their own typical portion sizes. This design methodology was originally selected with the intent of being quick, easy-to-administer, and minimize athlete/participant burden (i.e., such that the questionnaire could be completed in a field setting in ~10 minutes). A review focusing on the design, utilization and validation of FFQs, however, found that FFQs that are interviewer-administered and which allow subjects to report their own portion sizes are more likely to yield a correlation with their reference method [36]. In retrospect, the ability to enter typical rather than standardized portion size may be particularly important for a FFQ designed for active/athletic populations with varying energy requirements and eating habits. 

Despite quantitative differences between the FFQ and other methods, most previous studies have found weak-to-moderate correlations between FFQ-estimates of vitamin D and serum 25(OH)D concentration when comparisons were made over the winter months; in these studies, winter was specifically selected to avoid interference with endogenous vitamin D sources [27,32,33]. To our knowledge, however, only one published study has evaluated the relationship between a FFQ and markers of status in athletes. In this study, Fitzgerald et al. [20] found a strong association between 25(OH)D concentration and total vitamin D intake via the FFLQ in a relatively homogenous group of male hockey players. This correlation, however, appeared to be largely mediated by multivitamin and supplement intake [20]. 

Our lack of an association between vitamin D intake from food or food plus supplements and vitamin D status in the fall and spring was somewhat expected. In fact, the only notable correlation during these seasons was the weak associations between vitamin D intake from supplements and vitamin D status in the fall. Our lack of a direct association between intake and status in winter, however, was somewhat surprising, based on previous studies in non-athletes. These findings (which were weakly indirectly correlated) may be explained by the aforementioned shortcomings of the FFLQ and/or biological factors, including the potential buffering of serum 25(OH)D from adipose tissue (or skeletal muscle) stores over the winter months when cutaneous synthesis does not occur [37,38]. An additional shortcoming of the FFLQ may be the 3-month recall period, which is considerably longer than the 15-day half-life of 25(OH)D in circulation [35]. Furthermore, the biggest and most consistent predictor of status across all seasons (accounting for 10.5%–22.2% percent of the variation) was BMI. The inverse association between BMI and serum 25(OH)D has been observed previously in both athletic [39] and non-athletic [40,41] populations, with both body fat [11] and skeletal muscle thought to serve as vitamin D storage sites [42]. The extent to which these tissues buffer serum 25(OH)D during winter and other times of minimal UVB exposure, however, is not well understood. Thus, use of serum 25(OH)D concentration as a biomarker for FFQ validation has its limitations due to influence by BMI and factors other than diet.

To our knowledge, this current study is the first that has attempted to develop and validate a questionnaire that evaluates both sunlight (or UVB) exposure and diet. One recent study in a non-athletic population living in Denmark was able to validate a short-term (7-day) sun exposure questionnaire against serum 25(OH)D concentration [43]. Including UVB exposure questions as part of a FFQ seems to be imperative because factors such sun exposure and tanning bed use affect cutaneous synthesis [2,44] and may have a greater effect on vitamin D status than dietary sources [2,17], at least during warm, sunny seasons. Our attempts identified a consistent relationship between circulating 25(OH)D and reported tanning bed use in the fall and spring, and with leisure time spent outside (between 10:00 AM and 3:00 PM) in the fall using regression models that also accounted for BMI. The FFLQ, however, was unable to account for weather (cloud cover, etc.), clothing, and skin pigmentation during reported exposure. This, along with use of “leisure-time” rather than “training time”, may have led to some collection inaccuracies. Additional significant associations, which included sunscreen use in the fall and spring, may serve more as a marker of sunlight exposure. Properly applied sunscreen is known to decrease vitamin D synthesis [14], yet those who go outside more frequently also report more frequent sunscreen use. The same may be said for reported tanning bed use serving as a marker of outdoor sun exposure and desire to tan. While increases in serum 25(OH)D are probable with tanning bed use [44], many commercial beds do not emit UVB light. 

Results of the current analysis also point to possible shortcomings of the FFLQ that could be modified for future use. The shortcomings may be two-fold; an inability to accurately estimate vitamin D intake, and an inability to accurately estimate external factors like UVB exposure. While the limitations of estimating vitamin D intake using FFQ-methodology have previously been discussed, recent papers have highlighted more general errors associated with vitamin D content of foods listed in the USDA National nutrient data base. Holden and Lemar [45] have suggested that this database is incomplete, imprecise, and not inclusive of alternate forms of vitamin D in food including preformed 25(OH)D found in meat [46] and vitamin D2 found in brand-specific supplemental foods. This may be a significant omission, because dietary 25(OH)D has five times the biological activity of vitamin D3 [47]. Concerning estimation of UVB exposure, it is also likely the FFLQ could not fully account for our athletes’ exposure to sunlight and UVB radiation in sunny months. 

While this study provides important information addressing the difficulty of vitamin D intake assessment and vitamin D-specific questionnaire validation, it also has several limitations. One is that Study 1 and 2 were done with different subjects from different populations. The population of Study 2 was about 11 years older on average than that of Study 1, and consumed 155, 398, and 210 IU/day less in the fall, winter, and spring, respectively, most likely due to the higher caloric intake and dairy product consumption in the athletes compared to the healthy active subjects. A second is the lower number of data points available in athletes in the winter compared to the fall and spring in Study 1. Given the importance of assessing vitamin D intake and sun exposure habits in athletic and active populations, however, future revisions of the FFLQ will a) expand and update the list of vitamin D containing foods; b) allow for report of actual vs. standardized portion sizes; c) divide reported outside sun exposure into training and non-training leisure time; and d) link questions about clothing and sunscreen use to outdoor exposure. Delivery of an interviewer-administered questionnaire, use of a photographic album [23] during questionnaire completion, inclusion of meat products, and shortening of the FFLQ recall period (i.e 30 days vs. 3 months) to enhance recall and better reflect the 3-week half-life of serum 25(OH)D)) are also considerations for future modifications. 

## 5. Conclusions

The current study demonstrates that the FFLQ’s estimated vitamin D intake was not directly correlated with either serum 25(OH)D or estimated vitamin D intake from 7-day food diaries. The shortcomings of this questionnaire could be due to the limitations of dietary intake methodology in general to accurately estimate vitamin D content in food due to inaccuracies and omissions in the nutrient databases, and due to the errors associated with estimating sunlight exposure. This makes validation of a FFQ to assess vitamin D status difficult. Nevertheless, the availability of a valid questionnaire is important for assessing the risk and etiology of vitamin D insufficiency and deficiency in athletic and active populations, and the relationship of status to athletic performance. 

## Figures and Tables

**Table 1:** Characteristics of Division I Collegiate Athletes (Study 1).

	Fall (*n* = 86)	Winter (*n* = 49)	Spring (*n* = 67)
Male:Female	54:32	30:19	44:23
Race/Ethnicity (C, A, H, B)	78/3/2/3	45/2/1/1	60/2/1/4
Age (years)	20.2 ± 1.5	20.4 ± 1.7	20.6 ± 1.6
BMI (kg/m^2^)	23.8 ± 3.8	24.1 ± 4.2	23.9 ± 3.7
Sport (Indoor/Outdoor)	58/28	26/23	49/18
25(OH)D (ng/mL)	42.4 ± 15.5	27.9 ± 9.0	35.0 ± 15.5
Total Vitamin D Intake (IU/day)	800 ± 820	1236 ± 1131	727 ± 977
Vitamin D from Food Only (IU/day)	341 ± 228	584 ± 593	393 ± 616
Vitamin D from Supplements Only (IU/day)	459 ± 777	653 ± 987	334 ± 764
Leisure Time Outside in the Sun (h/day)	0.8 ± 0.7	0.7 ± 0.5	0.6 ± 0.6
Tanning Bed Use (h/day)	0.0008 ± 0.004	0.0002 ± 0.001	0.001 ± 0.004
Total UV Exposure (h/day)	0.83 ± 0.69	0.65 ± 0.52	0.61 ± 0.63

C, Caucasian; A, Asian; H, Hispanic; B, Black. Indoor Sports included Basketball, Swimming, and Wrestling; Outdoor Sports, Football, Soccer, Cross-country, Track & Field, Cheerleading.

**Table 2:** Association Between Serum 25(OH)D Concentration and Body Mass Index (BMI), Vitamin D Intake, and Markers of Sun Exposure (Study 1).

Correlation Coefficients	BMI	Vitamin D from Food Per Day	Vitamin D from Supplements Per Day	Total Vitamin D Per Day	Leisure Time Outside Per Day	Tanning Bed Use Per Day	Total UV Exposure Per Day	Frequency Sunscreen Use
Fall 25(OH)D (*n* = 86)	0.330**	−0.126	0.280**	0.133	0.130	0.176	0.133	0.226*
Winter 25(OH)D (*n* = 49)	−0.324*	−0.332*	0.152	−0.080	0.079	0.189	0.130	0.08
Spring 25(OH)D (*n* = 67)	0.472**	0.008	0.155	0.126	0.074	0.391**	0.096	0.479**

Spearman Rank Coefficients; * *p* < 0.05 **p < 0.01.

**Table 3:** Predictors of Serum 25(OH)D Concentration (Study 1)

Fall Model (R = 0.424, *p* < 0.001)		Winter Model (R = 0.315, *p* = 0.029)		Spring Model (R = 0.516, *p*<0.001)	
Predictor	Beta	Predictor	Beta	Predictor	Beta
BMI	−0.313	BMI	−0.315	BMI	−0.376
Tanning Bed Use	0.210			Tanning Bed Use	0.337
Outside Exposure	0.213				

Based on Backward Linear Regression Models with all variables except BMI entered as estimates of daily intake or daily UV exposure.

**Table 4:** Characteristics of Active Participants (Study 2).

Characteristic	Mean (*n* = 123)
Male:Female	60:63
Race/Ethnicity (C, A, H, B, AI, O)	112/5/3/1/1/1
Age (years)	31.3 ± 8.5
BMI (kg/m^2^)	24.8 ± 4.8
FFLQ Vitamin D Intake (IU/day)	186.4 ± 125.7
FFLQ Vitamin D Intake from Food Plus Supplements (IU/day)	341.5 ± 533.4
Food Diary Vitamin D Intake (IU/day)	148.5 ± 228.2
Food Diary Caloric Intake (kcal/day)	1871.0 ± 474.5

Data reported as mean ± standard deviation; C, Caucasian; A, Asian; H, Hispanic; B, Black, AI, American Indian or Alaskan Native, O, other.

**Figure A1:**
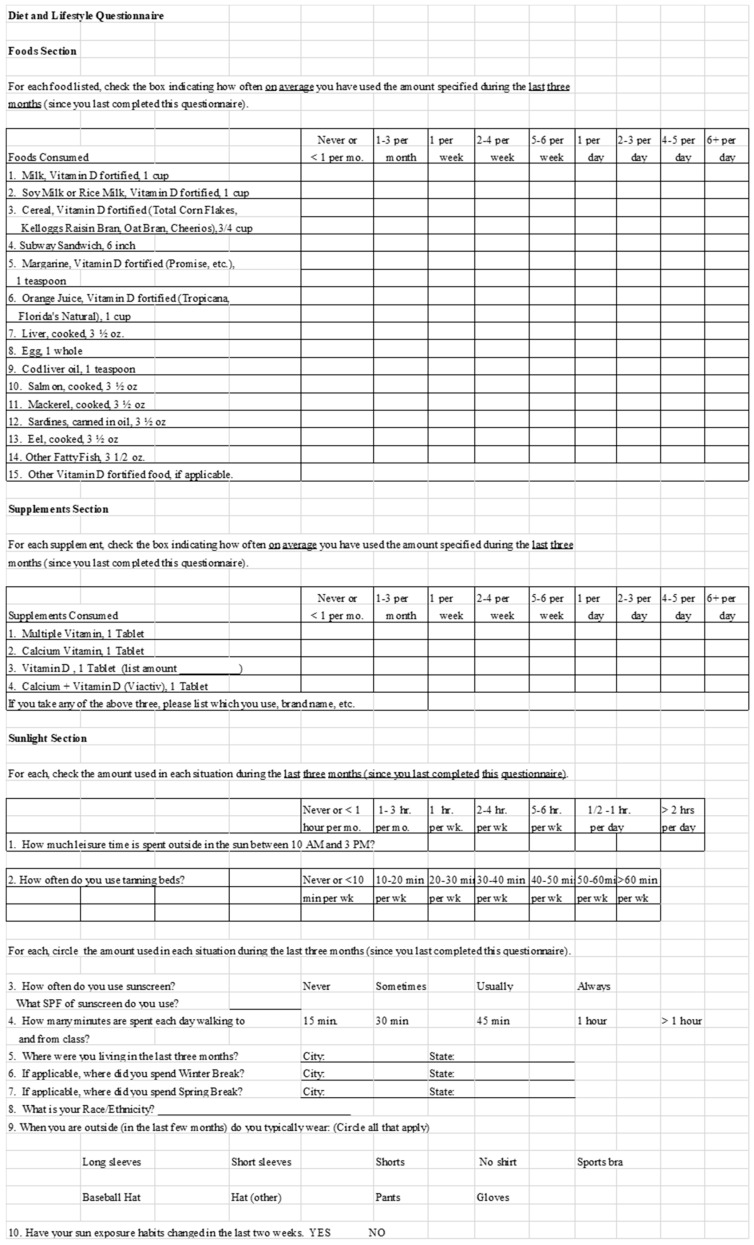
Vitamin D Food Frequency and Lifestyle Questionnaire.

## Supplementary Materials

The following are available online at https://www.mdpi.com/2072-6643/11/11/2732/s1, Figure S1: Food Diary Instruction and Design.

## Appendix A

The following is available below: Vitamin D Food Frequency and Lifestyle Questionnaire. Analysis program template and data available upon request.
