# Supplementary material for: Validation of a Vitamin D Specific Questionnaire to Determine Vitamin D Status in Athletes
Source: Nutrients. 2019 Nov 11;11(11):2732. doi: 10.3390/nu11112732 (PMC6893639; doi:10.3390/nu11112732)
Supplement: Supplementary file 1 [file nutrients-11-02732-s001.pdf]

nutrients MDPI

Supplemental Figure. Food Diary Instructions and Design

Researcher Initials______Subject # ______Week______ Date ______

24-HR DIETARY FOOD RECORD INSTRUCTIONS

1 **All foods** consumed should be recorded.

2 **Be very specific**. Make sure you include:

- the **type** of food
- the **amount** of each food
- the **preparation method** (i.e., fried, baked)
- the **brand name** of the food (if applicable)
- the **time** it was eaten
- the restaurant you ate it at (i.e., Subway, Applebees, Red Robin)

3 Record food consumption **after each meal/snack** instead of waiting until the end of the day.

4 **Save labels** from packages of food you eat and return them with your food record forms (this will greatly assist and enhance our analysis of your true nutrient intake).

5 Use nutrient descriptors (e.g., low-fat, low-carb, fat-free, light, reduced calorie, etc.).

6 **Include miscellaneous items** such as condiments (ketchup, salad dressing, mayonnaise, jams, creams, sugar), and chewing gum.

7 Record all **vitamins, minerals, herbals, supplements** (powders, shakes, bars, etc.).

8 Be careful about **"amount"** we would like these values as specific as possible. For packaged food providing "1 serving" is adequate, for homemade food please use standard measuring cups and spoons, weights and/or volumes. Examples: cup (c), Tablespoon (Tbs), teaspoon (tsp), fluid ounces (fl oz),weight ounces (oz), inches (in), milliliter (mL), gram (g), or centimeter (cm).

Day 1 **Date:_______**

TIME	DETAILED FOOD DESCRIPTION: BRAND NAME, RESTAURANT, METHOD OF PREPARATION, FLAVOR, CONDIMENTS, ETC.	AMOUNT
